# Utility of the bright and dark personality inventory in assessing personality pathology

**DOI:** 10.3389/fpsyg.2025.1608073

**Published:** 2025-10-09

**Authors:** Eunsil Cho, Yeoul Han, Kee-Hong Choi

**Affiliations:** ^1^School of Psychology, Korea University, Seoul, Republic of Korea; ^2^Mindeep Cognitive Behavioral Therapy Center, Seoul, Republic of Korea; ^3^KU Mind Health Institute, Korea University, Seoul, Republic of Korea

**Keywords:** five factor model, maladaptive personality trait, personality disorder, personality pathology, personality assessment

## Abstract

**Background:**

Contemporary approaches to personality pathology increasingly emphasize dimensional models, a shift reflected in recent diagnostic frameworks such as the DSM-5 Alternative Model for Personality Disorders (AMPD) and the ICD-11. Aligned with this perspective, the Bright and Dark Personality Inventory (BDPI), grounded in the five-factor model, was developed to dimensionally assess both general (“General 5”) and maladaptive (“Dark 5”) personality domains. This study focused on maladaptive personality traits and examined the incremental utility of the BDPI’s Dark 5 in identifying personality disorder (PD) tendencies in a nonclinical Korean sample.

**Methods:**

A total of 1,017 South Korean adults completed the BDPI, the Personality Inventory for DSM-5 – Short Form (PID-5-SF), and the Self-report Standardized Assessment of Personality Abbreviated Scale (SAPAS-SR). To examine convergent and incremental validity, we conducted Pearson correlations, squared semi-partial correlations, and hierarchical logistic regression analyses. In addition, independent samples t-tests were performed to assess group differences between individuals with and without PD tendencies.

**Results:**

The Dark 5 domains showed strong convergence with corresponding PID-5-SF traits, supporting their convergent validity. Negative Affectivity, Detachment, and Attention Difficulty predicted PD tendencies beyond the PID-5-SF, increasing explained variance by 9.7%. Egocentrism and Psychoticism contributed no unique variance, possibly due to suppression. Attention Difficulty, which includes obsessiveness, may partially reflect Anankastia-related traits.

**Conclusion:**

The BDPI’s Dark 5 may offer complementary value to existing trait-based assessments by capturing additional expressions of maladaptive personality traits. Further research should validate these findings in clinical populations and explore the measurement of Anankastia-relevant constructs.

## 1 Introduction

Personality disorders (PDs) are characterized by persistent maladaptive and inflexible personality traits that cause significant distress or impairment in social, occupational, and other functional domains (American Psychiatric Association, 2013). Recently, a dimensional approach was adopted in the Alternative Model for Personality Disorders (AMPD) of DSM-5 and its text revision (DSM-5-TR) and in the ICD-11 PD framework. Both offer more comprehensive and empirically supported models for understanding personality pathology (Bach et al., 2017).

Among existing trait-based assessments, the Personality Inventory for DSM-5 (PID-5) is one of the most widely used and psychometrically validated tools. However, because it was developed based on the AMPD, the PID-5 does not conceptualize Anankastia as a separate trait domain (Kerber et al., 2022; Strus et al., 2021), despite its central role in the ICD-11 structure. Although anankastic features are represented at the facet level—primarily via rigid perfectionism (within Disinhibition; reverse-keyed) and perseveration (within Negative Affectivity)—some scholars have suggested that the absence of a distinct Anankastia domain may limit the extent to which the PID-5 captures the full scope of this construct.

Given the complexity and multi-layered nature of personality pathology, relying on a single instrument may not adequately capture the full range of maladaptive traits. Empirical findings support this view: for instance, Fowler et al. (2017) showed that the PID-5 accounted for variance in psychopathology beyond both five-factor model traits and categorical PD criteria, illustrating that different instruments, even when targeting overlapping constructs, can provide unique contributions. Similarly, Heath et al. (2018) emphasized that variations in item content, response scaling, and cultural adaptation across instruments can generate complementary information. Taken together, these findings suggest that employing multiple instruments can help broaden construct coverage and potentially enhance predictive accuracy.

The Bright and Dark Personality Inventory (BDPI) was developed to assess both general (“General 5”) and maladaptive (“Dark 5”) personality traits within the frameworks of the five-factor model and dimensional PD models. The Dark 5 are designed to capture maladaptive expressions of general traits, such as obsessiveness and rigidity reflecting over-control aspects of Conscientiousness. While the BDPI Dark 5 and the PID-5 assess substantially overlapping maladaptive trait domains, differences in item pools, scaling, and domain aggregation suggest that they might capture partially distinct aspects of personality pathology. In this context, the BDPI’s Dark 5 could provide perspectives that are complementary to those of the PID-5. This possibility is exploratory in nature and requires cautious empirical evaluation regarding any incremental value.

The present study therefore seeks to explore, on a preliminary basis, whether the BDPI’s Dark 5 domains might contribute modest, complementary information alongside the PID-5 in relation to identifying PD tendencies within a nonclinical Korean sample. Consistent with this scope, analyses were conducted at the domain level; facet-level adjudication was beyond the present design. To classify participants with elevated PD tendencies, we employed the Self-report Standardized Assessment of Personality–Abbreviated Scale (SAPAS-SR), a brief screening tool designed to capture general indicators of personality dysfunction.

## 2 Methods

### 2.1 Participants

The data for this study were originally collected by Kim et al. (2020) as part of a nationwide survey to validate the BDPI, approved by the local institutional review board. Participants were recruited through an online survey panel to ensure demographic diversity in terms of age, sex, and region. After providing consent, participants completed four personality inventories and a demographic questionnaire.

The final sample consisted of 1,017 Korean adults (*M* age = 34.06, *SD* = 8.06), classified into PD tendency (*n* = 362) and non-clinical (*n* = 655) groups using SAPAS-SR scores. Demographic characteristics of the two groups are summarized in Table 1.

**Table 1:** Demographic characteristics of the participants (*N* = 1,017).

Characteristics	PD tendency group (*n* = 362)	Non-clinical group (*n* = 655)	*t or* $\chi^{2}$
Age (year), *M* (SD)	33.41 (7.97)	34.42 (8.10)	1.91
Gender, *n* (%)			3.99^*^
Male	167 (46.10)	345 (52.7)	
Female	195 (53.90)	310 (47.3)	
Years of education, *M* (SD)	15.07 (1.99)	15.26 (1.85)	1.55
Marital status, *n* (%)			15.53^**^
Single	233 (64.4)	350 (53.4)	
Married	121 (33.4)	297 (45.3)	
Divorced	8 (2.2)	7 (1.1)	
Separated	0 (0)	0 (0)	
Other	0 (0)	1 (0.2)	
Years of service (Current), *M* (SD)	3.91 (5.15)	4.19 (5.25)	0.80
Years of service (Total), *M* (SD)	7.48 (6.64)	8.21 (6.88)	1.64
Changing jobs, *M* (SD)	1.83 (1.88)	1.68 (1.99)	−1.16
Occupation, *n* (%)			20.72^**^
Office worker	173 (47.8)	297 (45.3)	
Technical post	24 (6.6)	69 (10.5)	
Service worker	18 (5.0)	36 (5.5)	
Self employed	19 (5.2)	35 (5.3)	
Agricultural, forestry and fishery worker	1 (0.3)	1 (0.2)	
Homemaker	23 (6.4)	58 (8.9)	
Student	42 (11.6)	98 (15.0)	
Unemployed	36 (9.9)	31 (4.7)	
Other	26 (7.2)	30 (4.6)	
Household monthly income (10, 000 KRW), n (%)			28.26^**^
≤ 199	54 (14.1)	44 (6.7)	
200 ~ 299	67 (18.5)	108 (16.5)	
300 ~ 399	57 (15.7)	131 (20.0)	
400 ~ 499	70 (19.3)	118 (18.0)	
500 ~ 599	35 (9.7)	95 (14.5)	
600 ~ 699	22 (6.1)	52 (7.9)	
700 ~ 799	13 (3.6)	40 (6.1)	
800 ~ 899	15 (4.1)	28 (4.3)	
900 ~ 999	10 (2.8)	9 (1.4)	
≥ 1,000	22 (6.1)	30 (4.6)	

^*^p < 0.05, ^**^p < 0.01.

### 2.2 Measures

#### 2.2.1 Bright and dark personality inventory (BDPI)

The BDPI (Choi et al., 2018) is a 173-item self-report measure developed to assess both general and maladaptive personality traits. It comprises three sections: an 8-item impression management scale, an 80-item scale measuring five general personality factors (the “General 5”), and an 85-item scale assessing five maladaptive personality factors (the “Dark 5”). The impression management scale is designed to assess the tendency to present oneself in an overly favorable light; lower scores indicate greater defensiveness or denial of minor faults. The General 5 include Extraversion–Introversion (e.g., Vitality, Gregariousness, Assertiveness, Introversion), Agreeableness (e.g., Trust, Generosity, Altruism), Conscientiousness (e.g., Persistency, Perfectionism, Orderliness), Openness (e.g., Experience openness, Intellectual openness, Aesthetic openness), and Emotional Stability (e.g., Emotional awareness, Emotional acceptance, Emotional expression). The Dark 5 domains, which were the focus of the present study, include Detachment (e.g., Anhedonia, Suspiciousness, Isolation), Egocentrism (e.g., Narcissism, Histrionic, Manipulativeness, Callousness), Attention Difficulty (e.g., Impulsivity, Obsessiveness, Distractibility), Psychoticism (e.g., Eccentricity, Unattunedness, Rigidity), and Negative Affectivity (e.g., Anxiousness, Irritability, Inferiority, Dependency). All items are rated on a 4-point Likert scale ranging from 1 (strongly disagree) to 4 (strongly agree). Representative items from the Dark 5 include: “I must never let my guard down around others” (Suspiciousness), “I hide the truth if it benefits me” (Manipulativeness), “I get so caught up in minor details that I fail to complete tasks on time” (Obsessiveness), “I find it difficult to tolerate situations where there is no clear answer” (Rigidity), and “I have never felt like I was a worthwhile person” (Inferiority).

The BDPI has been psychometrically validated in two large successive Korean community studies (Lee et al., 2019; Kim et al., 2020). In the initial validation study, Lee et al. (2019) conducted confirmatory factor analyses (CFA) with a community sample of 600 adults, supporting a hierarchical structure of both the General and Dark 5 dimensions. Model fit indices for the General 5 were acceptable (CFI = 0.88, TLI = 0.88, RMSEA = 0.040), and the Dark 5 also showed good fit (CFI = 0.88, TLI = 0.88, RMSEA = 0.05). In a subsequent study, Kim et al. (2020) replicated these findings in a larger sample of 1,017 participants, reporting acceptable model fit indices for both the General 5 (CFI = 0.86, TLI = 0.83, RMSEA = 0.07) and the Dark 5 (CFI = 0.88, TLI = 0.84, RMSEA = 0.08). In the same study, domain-level internal consistency was high, with Cronbach’s *α* ranging from 0.85 to 0.96. For the General 5, *α* coefficients were 0.92 for Extraversion–Introversion, 0.71 for Agreeableness, 0.87 for Conscientiousness, 0.86 for Openness, and 0.83 for Emotional Stability. For the Dark 5, α values were.87 for Detachment, 0.90 for Egocentrism, 0.87 for Attention Difficulty, 0.85 for Psychoticism, and.89 for Negative Affectivity. Test–retest reliability over a four-week interval was also adequate, ranging from 0.52 to 0.85 for the General 5 and from.64 to.77 for the Dark 5. These findings provide robust evidence for the structural reliability and factorial validity of the BDPI.

For the purposes of this study, only the Dark 5 domains were analyzed, as they more directly reflect maladaptive traits associated with personality pathology.

#### 2.2.2 Korean version of the personality inventory for DSM-5—short form (PID-5-SF)

The PID-5 was developed by Krueger et al. (2012) to assess pathological personality traits in the DSM-5 Personality Disorder Trait Model. The short-form version (PID-5-SF), consisting of 100 items, was subsequently introduced by Maples et al. (2015) to provide a more efficient assessment of the five pathological personality domains. In Korea, Shin and Hwang (2016) translated and validated the PID-5, while Hong et al. (2018) validated the PID-5-SF.

The PID-5-SF measures five domains—Negative Affectivity, Detachment, Psychoticism, Antagonism, and Disinhibition—across 25 subscales. Responses are given on a 4-point Likert scale (0 = strongly disagree, 3 = strongly agree). In this study, the five domains demonstrated high internal consistency, with Cronbach’s alpha values of 0.94 (Negative Affectivity), 0.92 (Detachment), 0.94 (Psychoticism), 0.91 (Antagonism), and 0.92 (Disinhibition).

#### 2.2.3 Korean version of the self-report standardized assessment of personality abbreviated scale (SAPAS-SR)

The SAPAS-SR is a brief, self-administered screening tool for PDs (Germans et al., 2013). It comprises eight items, each rated using a dichotomous response format (yes = 1; no = 0). Choi et al. (2015) translated and validated the Korean version. In the present study, the SAPAS-SR was used to classify participants into PD tendency and non-clinical groups based on their total score. With a cutoff score of 4, the Korean version of SAPAS-SR demonstrated a classification accuracy of 67.2% for PD patients. The internal consistency of the Korean version was satisfactory, with a Cronbach’s alpha of 0.79 (Choi et al., 2015).

### 2.3 Procedures

This study conducted a secondary analysis of data originally collected as part of the BDPI validation project by Kim et al. (2020). Data collection was conducted in three phases. In Phase 1, 1,307 participants completed the full 173-item BDPI. To reduce response fatigue, Phase 2 was administered 2 days later, during which 1,066 respondents completed additional measures, including the SAPAS-SR, the Korean version of the PID-5-SF, and a demographic questionnaire. Four weeks later, a randomly selected subsample was invited to complete the BDPI again to assess test–retest reliability, and 187 participants completed this follow-up survey. Additionally, to obtain supplementary clinical data, a subset of participants scoring 4 or higher on the SAPAS-SR were invited to complete the Korean version of the Structured Clinical Interview for DSM-5 Personality Disorders (SCID-5-PD). Given the limited number of completed interviews (*N* = 40), these data were not analyzed in the present study; however, the participants were retained in the dataset.

### 2.4 Data analysis

Based on the SAPAS-SR classification, group differences in categorical variables were analyzed using chi-square tests, while independent samples *t*-tests were used to compare continuous variables between the groups. Pearson correlations were calculated to examine associations among SAPAS-SR scores, PID-5-SF domains, and BDPI Dark 5 traits. Squared semi-partial correlations were computed to assess the unique contribution of each BDPI domain beyond the PID-5-SF. Variance inflation factors (VIFs) were examined to assess multicollinearity. Hierarchical logistic regression analyses were conducted to evaluate whether the BDPI Dark 5 provided incremental validity over the PID-5-SF in predicting PD tendency status.

## 3 Results

### 3.1 Demographic characteristics

Descriptive statistics for demographic variables are presented in Table 1. Chi-square tests indicated significant group differences in gender, marital status, occupation, and income, whereas no significant differences were found for age, education, or years of service.

### 3.2 Pearson and squared semi-partial correlation analyses among dark 5, PID-5-SF, and SAPAS-SR

Table 2 presents the means, standard deviations, and correlation coefficients for all study variables. The total SAPAS-SR score showed significant positive correlations with the PID-5 and Dark 5 (*p* < 0.01). Among personality traits, Antagonism (PID-5) and Egocentrism (Dark 5) had weak but significant correlations with the SAPAS-SR (*r* = 0.27, *p* < 0.01; *r* = 0.14, *p* < 0.01). Negative Affectivity (PID-5, Dark 5) showed a strong correlation with the SAPAS-SR (*r* = 0.52, *p* < 0.01).

**Table 2:** Pearson correlation analysis among Dark 5, PID-5-SF, and SAPAS-SR (*N* = 1,017).

		1	2–1	2–2	2–3	2–4	2–5	3–1	3–2	3–3	3–4	3–5
	1. Total SAPAS-SR score	1										
PID-5	2–1. Detachment	0.44^**^	1									
	2–2. Antagonism	0.27^**^	054^**^	1								
	2–3. Disinhibition	0.45^**^	005^**^	0.78^**^	1							
	2–4. Psychoticism	0.39^**^	0.69^**^	0.74^**^	0.83^**^	1						
	2–5. Negative Affectivity	0.52^**^	0.83^**^	0.69^**^	0.88^**^	0.78^**^	1					
Dark 5 of BDPI	3–1. Detachment	0.31^**^	0.68^**^	0.33^**^	0.49^**^	0.49^**^	0.57^**^	1				
	3-2. Egocentrism	0.14^**^	0.21^**^	0.61^**^	0.44^**^	0.47^**^	0.33^**^	0.41^**^	1			
	3-3. Attention Difficulty	0.45^**^	0.47^**^	0.40^**^	0.61^**^	0.51^**^	0.58^**^	0.56^**^	0.50^**^	1		
	3-4. Psychoticism	0.33^**^	0.48^**^	0.41^**^	0.55^**^	0.61^**^	0.53^**^	0.65^**^	0.61^**^	0.68^**^	1	
	3-5. Negative Affectivity	0.52^**^	0.60^**^	0.32^**^	0.58^**^	0.51^**^	0.69^**^	0.70^**^	0.40^**^	0.76^**^	0.69^**^	1
	M	3.11	1.96	1.89	1.87	1.70	1.97	2.24	2.08	2.29	2.15	2.28
	SD	1.74	0.59	0.50	0.50	0.57	0.51	0.43	0.40	0.41	0.39	0.42

^**^p < 0.01.Column headings correspond to the variables listed in the leftmost column (e.g., 1 = Total SAPAS-SR score, 2-1 = PID-5 Detachment).

To evaluate the unique predictive utility of the BDPI Dark 5 domains beyond the PID-5-SF, squared semi-partial correlations were calculated using SAPAS-SR scores as the outcome. After statistically controlling for the five PID-5-SF domains, Negative Affectivity (*R^2^* = 0.03), Detachment (*R^2^* = 0.01), and Attention Difficulty (*R^2^* = 0.01) each accounted for small but distinct portions of unique variance. By contrast, Egocentrism (*R^2^* = 0.00) and Psychoticism (*R^2^* = 0.00) demonstrated minimal incremental contribution.

### 3.3 Dark 5 and PID-5-SF in distinguishing PD tendency groups

An independent samples t-test was conducted to examine group differences in personality traits between individuals with and without PD tendencies. As shown in Table 3, scores across all domains of both the PID-5-SF and Dark 5 were significantly higher in the PD tendency group (*p* < 0.001).

**Table 3:** Differences in personality trait scores between individuals with and without PD tendencies (*N* = 1,017).

		PD tendency group (*N* = 362)		Non-clinical group (*N* = 655)		
		*M*	SD	*M*	SD	*t*-test
PID-5	Detachment	2.06	0.48	1.79	0.48	−8.636^***^
	Antagonism	2.34	0.53	1.74	0.50	−17.798^***^
	Disinhibition	2.16	0.43	1.71	0.46	−15.155^***^
	Psychoticism	2.31	0.41	1.77	0.45	−19.389^***^
	Negative affectivity	1.97	0.58	1.55	0.51	−11.716^***^
Dark 5 of BDPI	Detachment	2.49	0.38	2.11	0.39	−15.194^***^
	Egocentrism	2.15	0.40	2.04	0.39	−4.244^***^
	Attention difficulty	2.50	0.37	2.17	0.38	−13.633^***^
	Psychoticism	2.33	0.37	2.06	0.37	−11.123^***^
	Negative affectivity	2.57	0.35	2.13	0.37	−18.420^***^

^***^p < 0.001.

### 3.4 Incremental validity of dark 5 over PID-5-SF and trait-specific logistic analyses

To examine potential multicollinearity between the PID-5-SF and Dark 5 domains, VIFs were calculated prior to regression analysis. As shown in Table 4, all predictors exhibited VIFs below the conventional threshold of 10, indicating no serious multicollinearity. However, some domains—particularly Negative Affectivity (VIF = 8.81) and Disinhibition of PID-5-SF (VIF = 7.83), as well as Egocentrism (VIF = 6.02), Negative Affectivity (VIF = 6.89), Detachment (VIF = 5.48), and Attention Difficulty of Dark 5 (VIF = 5.18)—showed moderate levels of shared variance with other predictors, which warrants caution in interpreting their regression coefficients.

**Table 4:** Incremental predictive power of Dark 5 over PID-5-SF and multicollinearity diagnostics (*N* = 1,017).

	−2 Log Likelihood	Nagelkerke R^2^	*B*	*SE*(B)	*OR*	*Wald*	95% CI	VIF
Step1: PID-5-SF								
	1108.65	0.29						
Detachment			−0.84	0.26	0.43	10.55	[0.26, 0.72]	4.84
Antagonism			0.47	0.37	1.6	1.59^**^	[0.77, 3.31]	4.97
Disinhibition			−0.03	0.24	0.97	0.02	[0.61, 1.55]	7.83
Psychoticism			2.4	0.37	11.01	41.71	[5.32, 22.81]	4.21
Negative Affectivity			−0.84	0.26	0.43	10.55^***^	[0.26, 0.72]	8.81
Step 2: PID-5-SF + Dark 5								
	1010.98	0.39						
Detachment^a^			0.26	0.29	1.3	0.83	[0.74, 2.29]	
Antagonism^a^			0.3	0.35	1.34	0.7	[0.67, 2.68]	
Disinhibition^a^			−0.27	0.42	0.77	0.4	[0.34, 1.74]	
Psychoticism^a^			0.26	0.28	1.29	0.83	[0.75, 2.24]	
Negative affectivity^a^			0.73	0.45	2.08	2.7^*^	[0.87, 4.99]	
Detachment^b^			−0.78	0.33	0.46	5.46^**^	[0.24, 0.88]	5.48
Egocentrism^b^			−1.02	0.36	0.36	8.18^**^	[0.18, 0.73]	6.02
Attention difficulty^b^			1.34	0.34	3.8	15.01^***^	[1.94, 7.48]	5.18
Psychoticism^b^			−0.31	0.37	0.74	0.69	[0.36, 1.52]	4.38
Negative affectivity^b^			2.66	0.43	14.26	38.65^***^	[6.17, 32.95]	6.89

Each domain of Dark 5	−2 Log Likelihood	Nagelkerke R^2^	*B*	*SE*(B)	*OR*	*Wald*	95% CI
Detachment	1257.18	0.12	1.60	0.18	4.96^***^	82.88	[3.51, 6.70]
Egocentrism	1337.80	0.02	0.65	0.17	1.91^***^	15.04	[1.38, 2.63]
Attention difficulty	1156.73	0.24	2.65	0.22	14.20^***^	145.48	[9.23, 21.86]
Psychoticism	1253.40	0.13	1.76	0.19	5.84^***^	85.34	[4.01, 8.49]
Negative affectivity	1075.85	0.32	3.23	0.24	25.36^***^	182.51	[15.87, 40.55]

SE, standard error; OR, odds ratio; CI, confidence interval; VIF, Variance Inflation Factor.

^*^p < 0.05, ^**^p < 0.01, ^***^p < 0.001. ^a^PID-5-SF domains; ^b^BDPI Dark 5 domains.

Hierarchical logistic regression analysis was conducted to examine whether the Dark 5 contributes incremental predictive utility beyond the PID-5-SF in distinguishing individuals with PD tendencies, as classified by the SAPAS-SR cutoff score (Table 4). Scores of 4 or higher on the SAPAS-SR were coded as 1 (PD tendency), whereas scores below 4 were coded as 0 (non-clinical).

In Step 1, the model including only the five PID-5-SF domains was statistically significant, accounting for 29.1% of the variance in PD tendency (−2LL = 1108.65, Nagelkerke *R^2^* = 0.29). In this model, Antagonism (*B* = 0.30, *p* < 0.01) and Negative Affectivity (*B* = 0.73, *p* < 0.001) significantly predicted PD tendency status.

In Step 2, the Dark 5 domains were added to the model. The expanded model showed improved model fit (−2LL = 1010.98), with the explained variance increasing to 38.8% (Nagelkerke *R^2^* = 0.39), indicating that the BDPI accounted for additional variance in PD tendency beyond the PID-5-SF. In the final model, five variables significantly predicted PD tendency status: Negative Affectivity of PID-5-SF (*B* = 0.55, *p* < 0.05), Detachment (*B* = 0.94, *p* < 0.01), Egocentrism (*B* = −0.93, *p* < 0.01), Attention Difficulty (*B* = 1.61, *p* < 0.001), and Negative Affectivity (*B* = 1.76, *p* < 0.001) of the BDPI.

Given the relatively high VIF values observed among some predictors, an additional set of logistic regression analyses was conducted to examine the predictive utility of each Dark 5 trait individually. This approach aimed to evaluate the classification accuracy and statistical contribution of each trait while minimizing potential confounding due to multicollinearity. All five models were statistically significant (Table 4), with explanatory power ranging from 2 to 32%. Classification accuracy for PD tendency varied across traits, with Negative Affectivity showing the highest accuracy (73.2%), followed by Attention Difficulty (70.2%), Psychoticism (67.6%), Detachment (66.2%), and Egocentrism (61.8%). Higher levels of all five traits were significantly associated with an increased probability of PD tendency (*B* = 1.91 to 25.36, *p* < 0.001).

## 4 Discussion

Using data from 1,017 community-dwelling Korean adults, we examined the empirical utility of the BDPI’s Dark 5 in assessing PD tendencies within a dimensional trait framework. Several main findings emerged. Within the limits of the present design, these results speak to domain-level incremental utility; facet-level differentiation was not directly tested.

First, independent samples t-tests confirmed that individuals with PD tendencies scored significantly higher across all Dark 5 and PID-5-SF domains. This suggests that the Dark 5 may help differentiate between individuals with and without elevated personality pathology in a community sample. These results align with previous research highlighting elevated pathological traits in PD tendency groups (Bach et al., 2018; Lugo et al., 2019; Rowiński et al., 2019). Thus, the BDPI’s Dark 5 may hold utility as a screening measure for identifying personality pathology in nonclinical settings.

Second, the Dark 5 domains demonstrated strong correlations with both the SAPAS-SR and their corresponding PID-5-SF domains. Given that the PID-5 has been extensively validated as a measure of pathological personality traits (Anderson et al., 2018; Fowler et al., 2017), these results provide support for the BDPI’s convergent validity within a dimensional model of personality pathology.

Notably, semi-partial correlation analyses controlling for the PID-5-SF domains revealed that the BDPI’s Negative Affectivity, Detachment, and Attention Difficulty accounted for additional unique variance in PD tendencies. Hierarchical logistic regression analyses further confirmed these results, revealing an additional 9.7% of explained variance when the Dark 5 domains were added to the PID-5-SF model—representing a small-to-moderate effect size (Cohen, 1988). Taken together, these findings tentatively suggest that the BDPI may contribute modest incremental variance in PD tendencies beyond the PID-5-SF. In particular, domains such as Negative Affectivity, Detachment, and Attention Difficulty may point to maladaptive expressions that could merit further exploration alongside established measures. For example, Attention Difficulty emerged as a meaningful predictor of PD tendencies. As previously noted, the obsessiveness facet within this domain reflects perfectionistic tendencies, akin to rigid perfectionism described in the PID-5. It also captures behavioral patterns such as excessive focus on minor details and lowered task efficiency. These additional features may align with trait expressions relevant to Anankastia, although further research is needed to clarify their specific contribution.

Interestingly, the Dark 5’s Psychoticism did not contribute unique predictive value in either semi-partial or logistic regression analyses, despite including the rigidity facet that was hypothesized to reflect ICD-11 Anankastia. One possible explanation is that the BDPI’s Psychoticism domain also includes traits such as perceptual dysregulation and odd beliefs (captured by the facets of eccentricity and unattunedness), which may have diluted the predictive signal of cognitive rigidity in community samples. In addition to these content-related factors, Psychoticism also exhibited a high VIF value, indicating substantial multicollinearity with other predictors, which may have further limited its statistical contribution. This pattern is consistent with our conceptual cross-walk: anankastic features are distributed across the BDPI’s obsessiveness (Attention Difficulty) and rigidity (Psychoticism) facets, rather than concentrated within a single domain. Similar to the PID-5-SF, the BDPI does not provide Anankastia as a standalone domain, which highlights a broader structural challenge in trait-based models. Future research should explore whether psychometrically isolating Anankastia-relevant traits—such as rigidity and obsessiveness— may clarify how these traits relate to the ICD-11 framework, where Anankastia is emphasized as a distinct domain. Given that this study did not conduct facet-level analyses, these interpretations remain provisional. More fine-grained analyses are needed to determine whether specific facets contribute differentially to PD tendencies.

In addition, Egocentrism showed a negative association with PD tendency in hierarchical logistic regression, despite being positively associated in single-predictor logistic models. This may also have been partly attributable to its elevated multicollinearity. This suppression effect, also observed in the absence of unique variance in semi-partial correlations, suggests complex interdependencies among the Dark 5 domains and calls for cautious interpretation in multivariate settings.

This study has several limitations. First, PD tendency was assessed using SAPAS-SR, a brief screening tool rather than a diagnostic instrument, potentially limiting classification precision. Because the SAPAS-SR captures general personality dysfunction rather than categorical diagnoses, associations with the BDPI may reflect global maladaptive tendencies rather than disorder-specific liability. Second, as the study was conducted using a non-clinical Korean sample, further research is needed to evaluate the BDPI’s reliability, validity, and structural properties across both clinical populations and diverse cultural contexts. Third, the difference in item counts between the BDPI (173 items) and the PID-5-SF (100 items) may have influenced their relative explanatory power. It is presumed that the short form of the PID-5 was selected in the original design by Kim et al. (2020) to minimize participant fatigue; however, future studies should consider employing the full-length versions of both instruments for a more balanced comparison. Fourth, the present study evaluated incremental utility at the domain level rather than the facet level. We did not directly contrast facet-level proxies for anankastic features across the PID-5 (rigid perfectionism, perseveration) and the BDPI (obsessiveness, rigidity). Future work should preregister and adequately power facet-level models that explicitly address domain–facet variance partitioning and multicollinearity, in order to test whether specific facets differentially contribute to PD tendencies. Fifth, although the BDPI assesses both general and pathological personality traits, the present study focused exclusively on the Dark 5. The General 5 (e.g., Extraversion-Introversion, Agreeableness, Conscientiousness, Openness, Emotional Stability) also provide valuable information about individuals and may complement maladaptive traits. Therefore, future studies should examine the interactions between general and maladaptive personality dimensions. Finally, all data were collected via self-report measures, which are subject to biases such as social desirability, self-awareness limitations, and response styles. Subsequent studies could address these limitations by incorporating informant reports or clinician-administered tools, such as the SCID-5-PD, to further evaluate the BDPI’s diagnostic utility.

## Data Availability

The data analyzed in this study is subject to the following licenses/restrictions: the dataset analyzed in this study was originally collected under IRB-approved protocols by a separate research team. As the current study involved a secondary analysis of de-identified data, additional ethical approval was not required. Due to privacy and ethical considerations, the dataset is not publicly available and was shared under restricted access by a separate research team. Requests for access can be initially directed to the corresponding author of this article at kchoi1@korea.ac.kr, who can forward inquiries to the original data custodians.
